# Continuous Activity Monitoring Using a Wearable Sensor in Dogs with Osteoarthritis: An Exploratory Case Series

**DOI:** 10.3390/ani15182639

**Published:** 2025-09-09

**Authors:** Carina Sacoor, Sara Leitão, Carolina Domingues, Joana Babo, Cátia M. Sá, Ricardo Cabeças, Felisbina L. Queiroga

**Affiliations:** 1 Vasco da Gama Research Center (CIVG), Vasco da Gama University School (EUVG), 3020-210 Coimbra, Portugal; 2 Department of Veterinary Sciences, School of Agrarian and Veterinary Sciences, University of Trás-os-Montes e Alto Douro (UTAD), 5000-801 Vila Real, Portugal; 3 Maven Pet Inc., Wilmington, DE 19801, USA; sara.leitao@maven.pet (S.L.); carolina@maven.pet (C.D.); joana@maven.pet (J.B.); 4 Centro Veterinário VetCura, 4050-145 Porto, Portugal; 5 Raisevet, 4050-145 Porto, Portugal; 6 Animal and Veterinary Research Center (CECAV), University of Trás-os-Montes and Alto Douro, 5001-801 Vila Real, Portugal; 7 Associate Laboratory for Animal and Veterinary Sciences (AL4AnimalS), 1300-477 Lisboa, Portugal

**Keywords:** accelerometry, osteoarthritis, real-time monitoring, smart collars, wearable sensors

## Abstract

Canine osteoarthritis (OA) is a chronic, progressive disease that impacts mobility and welfare, often with subtle clinical signs that fluctuate over time. This exploratory case series evaluated the potential of a wearable sensor system (Maven Pet AI System) to detect real-time deviations in activity and rest patterns in dogs with OA under home-based conditions. Five client-owned dogs were monitored over periods ranging from 56 to 126 days, generating longitudinal data on activity and rest patterns. Nine clinically relevant events were identified: seven OA-related flare-ups and two non-orthopedic health issues. In eight of these events, deviations in activity profiles were temporally aligned with symptom onset, therapeutic response, or recovery. Statistically significant changes were observed in six out of nine events, particularly in the Active and Excited categories, while visual trend analysis revealed clinically relevant deviations even in the absence of statistical significance. In one case, decreased activity preceded owner recognition, suggesting potential for early detection. Sensor data also contextualized episodes of overexertion and non-orthopedic conditions, such as pruritus and gastroenteritis. Owner and clinician feedback indicated high usability and perceived clinical value. Despite the small sample, these findings suggest that continuous sensor-based monitoring may complement conventional evaluations and support earlier, more individualized OA management in real-world settings. Further studies are needed to validate and expand these preliminary observations.

## Simple Summary

Osteoarthritis is a long-term, painful condition that affects the quality of life of dogs. Early signs are often subtle and may go unnoticed. In this study, five dogs with osteoarthritis wore a smart collar that tracked their activity and rest over several weeks. During the monitoring period, nine health events were identified, most of them related to joint pain. In eight of these events, the collar recorded changes in activity patterns that matched the start, progression, or recovery of the event. While some activity changes were not significant, the patterns observed still provided useful clinical insights. These observations may suggest the usefulness of continuous monitoring through smart collars as a tool to support the management of osteoarthritis in dogs.

## 1. Introduction

Osteoarthritis (OA), a degenerative joint disease, is a chronic, progressive condition in dogs [1] characterized by the degradation of articular cartilage, subchondral bone remodeling, and low-grade synovial inflammation, and represents a leading cause of chronic pain [2].

Canine OA can negatively impact the overall welfare of dogs [1] as it causes reduced mobility, chronic pain and a progressive decline in quality of life [3]. Clinical signs often include persistent lameness, joint stiffness and reluctance to exercise [4]. Progressively, these mobility restrictions lead to secondary complications such as muscle atrophy, worsening obesity, and further degeneration of the joints. Behavioral alterations such as fearfulness, withdrawal from interaction and decreased social behavior reflect the emotional burden of chronic pain [3]. OA may also disrupt sleep patterns, leading to reduced duration and continuity of night-time rest, which can further compromise the animal’s overall welfare [5]. In addition, OA can negatively affect owner-dog interactions, with reduced walking activity and diminished enjoyment of shared routines [6]. These physical, emotional, and social consequences [3] underscore the importance of early detection, comprehensive management, and targeted intervention strategies [4].

Owner-reported outcome measures, such as pain scoring systems and mobility questionnaires, have gained prominence in both clinical and research settings as adjunctive tools for evaluating functional impairment and quality of life. Validated instruments such as the Canine Brief Pain Inventory [7] and the Liverpool Osteoarthritis in Dogs questionnaires [8] provide insights into severity and impact of pain perception from the owner’s perspective. While these tools enhance the understanding of patient-reported outcomes, they remain susceptible to owner bias and observational inconsistencies [9]. Still, strong correlations between multiple clinical methodology instruments indicate that they provide similarly relevant information and using multiple instruments may help mitigate individual biases and contribute to a more comprehensive and reliable evaluation of the complex paradigm of OA [10].

In dogs with OA, clinical evaluations at isolated time points may fail to capture daily fluctuations in mobility, pain or behavior. Additionally, clinical signs can be masked by stress or excitement in unfamiliar environments, often leading to underestimation of disease severity [11]. These challenges are compounded by inconsistent owner recognition of early signs, variable adherence to veterinary recommendations, and communication barriers. Moreover, decision-making is often asynchronous: owners follow a personal, interpretive process to decide when and whether to seek care, whereas veterinarians base their actions on clinical signs and diagnostic criteria once the animal is presented. This divergence in timing and perspective may delay intervention and contribute to misaligned expectations regarding diagnosis and treatment [12].

Wearable systems have recently been explored for the continuous assessment of locomotor function in dogs with OA [13,14,15]. These devices typically incorporate inertial measurement units, including accelerometers and gyroscopes, enabling objective, non-invasive quantification of activity levels, postural transitions and gait dynamics, under naturalistic conditions [16,17,18,19,20,21,22,23]. Such systems align with emerging perspectives in healthcare toward continuous health monitoring outside traditional clinical environments, supporting more timely and data-driven decision-making [24]. By offering an alternative pathway to clinical diagnosis, these wearable technologies have been increasingly recognized as part of future strategies for proactive, data-driven healthcare [25].

In this study, we investigated the use of a wearable remote monitoring system (Maven Pet AI System), combining accelerometry and gyroscopic sensing, for continuous activity assessment in dogs diagnosed with OA. Unlike previous short-term or group-based studies [13,14,15,26,27,28,29], our approach focused on individualized, long-term tracking in a real-world setting. Activity data were aligned with real-life clinical events and interpreted in conjunction with owner-reported information and veterinary records. This prospective and exploratory study followed a descriptive design aimed to identify subtle, time-specific deviations in activity patterns that might otherwise go unnoticed. Our goal was to generate preliminary insights into feasibility, clinical relevance and interpretability of continuous sensor-based monitoring in client-owned dogs with OA.

## 2. Materials and Methods

All procedures complied with the Portuguese legislation for the protection of animals used for scientific purposes (i.e., Decree-Law no. 113/2013, of 7 August 2013), which transposes European legislation (i.e., Directive 2010/63/EU of the European Parliament and of the Council, of 22 September 2010). All participants, owners and clinicians provided written informed consent.

### 2.1. Study Setting and Participants

The study was conducted between 30 November 2023 and 5 April 2024 at the Centro de Reabilitação Animal das Oliveiras, Porto, Portugal. A total of 15 mixed-breed client-owned dogs were enrolled in the study, each with a confirmed clinical diagnosis of OA established by the veterinary team at the rehabilitation center. All enrolled dogs were under continuous clinical supervision throughout the study period. This supervision involved both remote and in-person follow-up by the attending veterinarian, when necessary, as well as remote monitoring by the Maven veterinary team, who contacted owners whenever relevant changes in activity were detected to clarify potential clinical or contextual causes.

Eligible dogs met the following inclusion criteria: preserved ambulatory function, residence in the same household as their owner, and consistent daily interaction. In addition, owners were required to be fluent in English, have access to an iOS device, and maintain a stable home internet connection. A minimum monitoring period of 56 days (1248 h) was also required. No restrictions were applied regarding concomitant diseases, sex, neutering status, age, or breed. Exclusion criteria included: severe mobility impairment rendering the dog non-ambulatory; insufficient daily interaction with the primary caregiver, preventing consistent home monitoring; and discontinuation of sensor use for more than seven consecutive days.

### 2.2. Monitoring System Description

**Figure 1:**
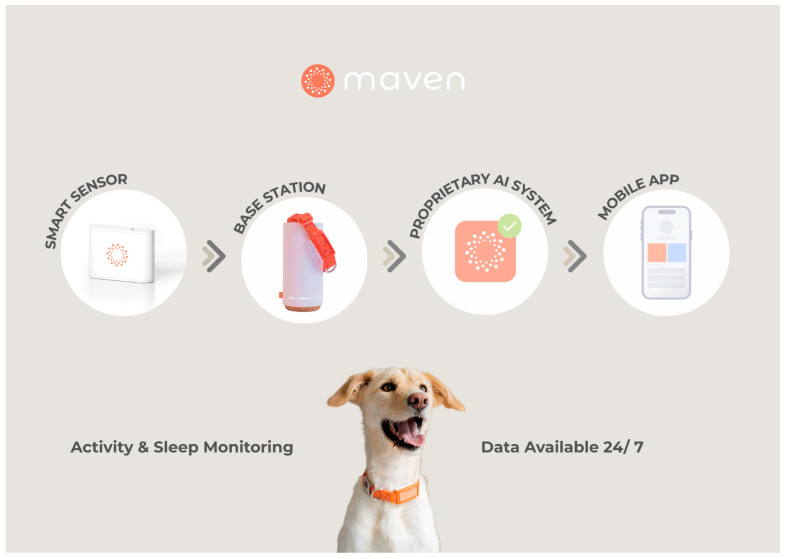
Overview of the Maven AI System. The Maven AI System enables continuous remote monitoring by collecting activity and sleep data through a smart sensor, transmitting it via a base station to a cloud infrastructure, where AI algorithms process the information and deliver real-time insights through a mobile interface.

The Maven Pet AI System, DE, USA, consisted of a collar-mounted motion sensor with a triaxial accelerometer and gyroscope (6-axis) capable of collecting real-time data on the animal’s physical activity and posture. The recorded data were transmitted automatically to a home-based synchronization station via wireless connection (Figure 1). The system also included an iOS-specific mobile application that allowed pet owners to access daily summaries, symptom tracking tools, and digital health checkups. Simultaneously, a cloud-based clinical dashboard enabled the veterinary team to remotely monitor health parameters, visualize trends over time, and respond to system-generated alerts based on deviations from the dog’s baseline. The sensor continuously monitored the dog’s movement and posture, capturing temporal high-resolution activity data. These data were processed in real time by embedded algorithms, enabling the automated classification of activity states relevant to both clinical interpretation and longitudinal health monitoring:

- Resting: prolonged periods of inactivity typically associated with sleep or deep rest.
- Quiet: awake but not in continuous motion, such as moving the head, chewing on a toy, licking, or standing still.
- Active: sustained, low-paced activity involving motion, such as walking or pacing.
- Excited: high-intensity activity, including trotting, running or jumping.
- Not resting: sum of Quiet, Active and Excited.

Additional data included the following: owner input, encompassing digital symptom journaling and standardized monthly health assessments consisting of 15-item questionnaires about relevant clinical indicators, including changes in gastrointestinal function, locomotion, respiratory effort, behavior, and dermatologic health.

### 2.3. Procedures

An onboarding process was conducted where dog owners received a comprehensive overview of all the application’s features. After informed consent, owners received the Maven kit and installed the mobile application. Data collection started upon activation of the sensor, which remained in place for a minimum of 8 weeks. For each dog, a personalized baseline was established during the first 14 days of use, assuming clinical and environmental stability. Each dog was monitored longitudinally, with data interpreted in reference to its own individualized baseline.

Daily sensor data were automatically synchronized to the cloud and analyzed byAI algorithms. Owners were requested to consistently log any symptoms they identified using the application and to answer remote digital health checkups. Pet owners agreed to be contacted weekly by a veterinarian from the study team to review system alerts and provide context for any observed deviations. Clinically relevant events, such as pain flare-ups, episodes of overexertion and other health issues, were identified through this process and compared to the activity and rest patterns detected by the system. When deviations occurred without a clearly associated medical cause, owners were contacted to explore potential non-clinical explanations, including environmental changes and alterations in household routines. Given the exploratory nature of the study, no data points associated with non-clinical events were excluded from analysis; these events were, however, considered qualitatively during the interpretation of individual cases (e.g., fireworks, home gatherings, travels, etc.).

### 2.4. AI-Vet Alert System and Clinical Access

The veterinarian in charge of the study (CMS) at the Centro de Reabilitação das Oliveiras, who was also responsible for case management, was granted access to the Vet Portal, a web-based platform where each dog’s daily health assessments were made available. These assessments were generated by integrating behavioral and physiological data, specifically activity and sleep, alongside clinical history and owner-reported observations via the Maven iOS application. System outputs included temporal trend graphs, raw parameter values, and algorithm-generated interpretations to support clinical evaluation and decision-making.

To facilitate efficient interpretation, a color-coded tagging system was employed to classify the animal’s daily status, and corresponding alerts were generated. In this context, an alert refers to an automated notification triggered when specific deviations from the dog’s usual activity or rest patterns exceed predefined criteria established by the algorithm. A green tag denoted parameters within expected limits. A yellow tag was assigned when minor deviations were detected that warranted closer monitoring. A red tag was used to highlight clinically significant deviations that could indicate a need for veterinary intervention. Tags were always accompanied by brief system-generated descriptors of the underlying triggers, such as atypical activity levels or disrupted sleep patterns. Alerts generated by the collar (yellow or red) were subsequently classified as events only when their occurrence was confirmed by the owner and the influence of external factors was excluded (e.g., fireworks, home gatherings, travel).

### 2.5. Usability and Perception Assessment

Feedback from dog owners was collected through a user experience questionnaire administered at two distinct time points: after the initial three weeks of system use and again during the final week of the study. In parallel, the veterinarian responsible for case management (CMS) completed a structured feedback form following the conclusion of the study, assessing the system’s clinical relevance, ease of integration, and impact on communication with owners.

### 2.6. Data Analysis Approach

All sensor-derived data were exported from the cloud platform in standardized tabular format as Microsoft Excel files. Subsequent data processing and statistical analyses were performed in R (version 4.3.3). For each monitored dog, daily activity summaries were calculated per category (Rest, Quiet, Active, Excited, and Not resting, defined as the sum of Active states), expressed as mean minutes per hour (±standard deviation). These summaries were used to construct individual activity profiles. Time-series plots with shaded standard deviations were generated to visualize temporal trends, covering a time window of five days before and five days after the end of each event period.

Each subject’s baseline was defined as a 14-day period of clinical and environmental stability, serving as a personalized reference for identifying metric fluctuations. In cases where a clinical event coincided with the beginning of the monitoring period, a new baseline was established following clinical stabilization. Observed patterns were qualitatively interpreted in the context of each animal’s individualized baseline and cross-referenced with owner-reported symptoms and clinical follow-up information.

Statistical comparisons focused on detecting changes in activity levels during predefined event periods, based on veterinary records and owner-reported information. As the data did not conform to a normal distribution, non-parametric methods were applied. Mann-Whitney U tests were used for pairwise comparisons between each dog’s baseline and the corresponding event period.

All statistical tests were performed individually per dog and per category, reflecting the exploratory nature of the study and the substantial inter-individual variability observed in responses to clinical events. A significance threshold of *p* < 0.05 was adopted.

## 3. Results

### 3.1. Study Population Overview

A total of five client-owned dogs with a confirmed diagnosis of OA were included in the final analysis, following the exclusion of ten subjects based on predefined criteria: eight due to insufficient owner-dog interaction that precluded consistent monitoring, and two due to sensor usage discontinuation exceeding seven consecutive days. All included dogs maintained ambulatory capacity throughout the study and completed the full remote monitoring protocol using the Maven system.

The monitoring period ranged from 56 to 126 days (mean 94.2 ± 23.6 days), resulting in 1248 to 2873 h of valid sensor-derived data per dog. All enrolled dogs had a confirmed diagnosis of OA. Comorbidities observed in the cohort included chronic ehrlichiosis with systemic involvement, skin allergies, degenerative valvular disease, and spondylosis. Key demographic, clinical, and monitoring characteristics for each enrolled subject are summarized in Table 1.

### 3.2. Clinical Characterization of Osteoarthritis in the Study Dogs

A concise clinical characterization of the included dogs, including main diagnosis, joints affected, OA severity, lameness grade, and changes observed during the study period, is summarized in Table 2. In this table, event classification refers to clinically documented episodes further detailed in Section 3.3.

In dogs D1, D2, D3, and D5, diagnosis was confirmed through clinical and radiographic evaluation. Radiographs of D1 showed bilateral shallow acetabula, remodeling of the femoral head-neck junctions, marked osteophyte formation along the acetabular rims, and subchondral sclerosis. In D2, radiographs revealed osteophyte formation along the acetabular rims, remodeling of the femoral head-neck junctions, and subchondral sclerosis. D3 radiographs demonstrated shallow acetabula with reduced femoral head coverage, remodeling of the femoral head-neck junctions, and periarticular osteophyte formation more pronounced on the left side, together with subchondral sclerosis of the acetabular roofs. In D5, remodeling of the femoral head-neck junction, periarticular osteophyte formation, and the presence of chondrocalcinosis in right hip joint were observed. In D4, OA secondary to immune-mediated polyarthritis caused by ehrlichiosis was diagnosed based on compatible clinical presentation and PCR confirmation. No follow-up imaging was performed during the study, as no new clinical indications warranted reassessment. All dogs were managed conservatively, with the aim of maintaining comfort and functionality in the context of chronic disease.

### 3.3. Event Classification and Distribution

When a system alert was generated, the study’s veterinary team contacted the owner to contextualize the deviation and verify whether observable changes in the dog’s behavior or mobility had occurred. Confirmed cases were formally classified as events; non-confirmed cases were classified as issues. During the monitoring period, a total of nine clinically relevant events were identified across five dogs. Although the primary focus of this study was the monitoring of OA-related events, additional non-orthopedic health episodes were also detected and retained for analysis, in line with the exploratory and observational design of the protocol. For clarity and comparative purposes, all events were systematically classified into two categories: (1) OA Flare-up Events (*n* = 7), comprising episodes of lameness, stiffness, or reduced willingness to move due to OA exacerbation (*n* = 4); transient mobility declines following identifiable peaks in physical activity (*n* = 2); and OA manifestations secondary to systemic conditions with inflammatory or immune-mediated mechanisms (*n* = 1); and (2) Non-Orthopedic Health Events (*n* = 2), including clinical episodes unrelated to OA, such as dermatological or gastrointestinal disturbances, that were nonetheless considered relevant for inclusion due to their potential to influence activity or rest patterns.

The distribution of events by category, dog identification, the duration of each event, and the respective case summary are detailed in Table 3.

In dogs D2 and D3, the originally intended 14-day baseline period did not precede the first clinical event. In both cases, the onset of the events 3 and 5 coincided with the first day of sensor use, and a new baseline was subsequently established following clinical stabilization.

### 3.4. Sensor Based Activity Analysis by Event Type

**Figure 2:**
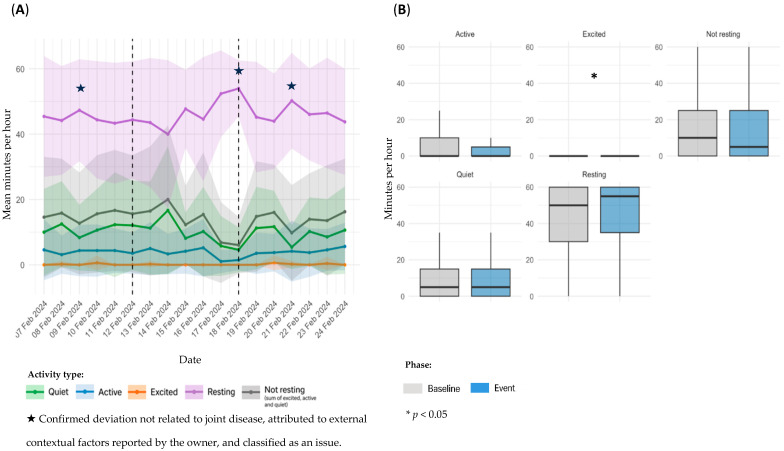
Sensor-derived activity data for dog D1, referring to Event 1; (**A**): temporal trends in mean daily activity (minutes per hour) ± SD across pre-event, event, and post-event periods; (**B**): comparison of activity metrics between individual baseline and event period.

**Figure 3:**
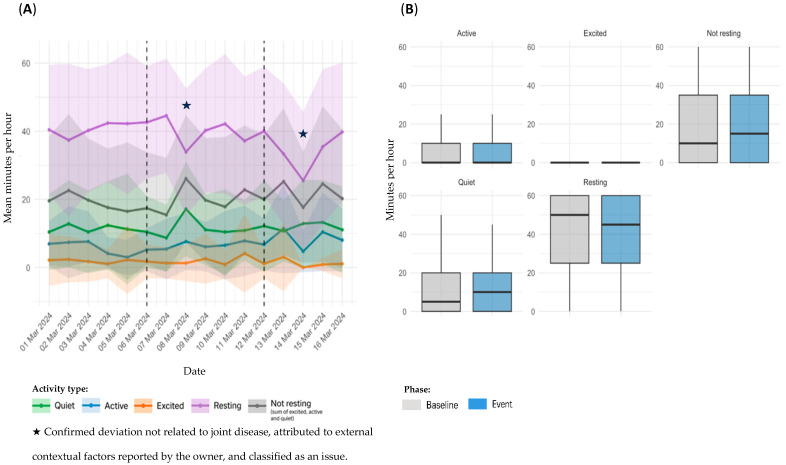
Sensor-derived activity data for dog D2, referring to Event 2; (**A**): temporal trends in mean daily activity (minutes per hour) ± SD across pre-event and post-event periods; (**B**): comparison of activity metrics between individual baseline and event period.

**Figure 4:**
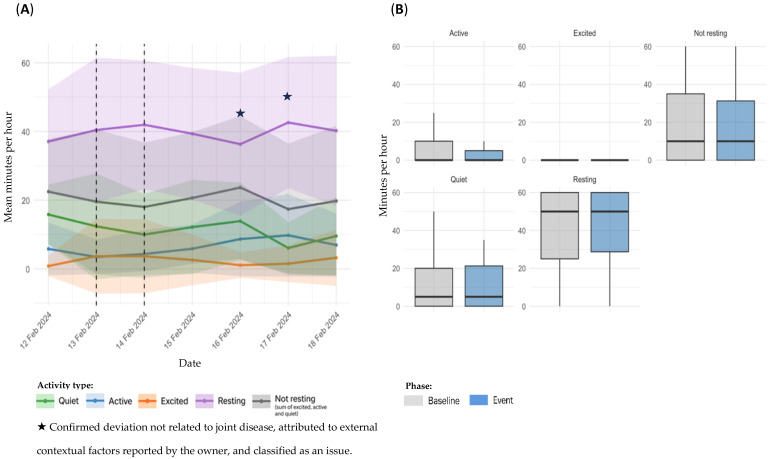
Sensor-derived activity data for D2, referring to Event 3; (**A**): temporal trends in mean daily activity (minutes per hour) ± SD across pre-event, event, and post-event periods; (**B**): comparison of activity metrics between individual baseline and event period.

**Figure 5:**
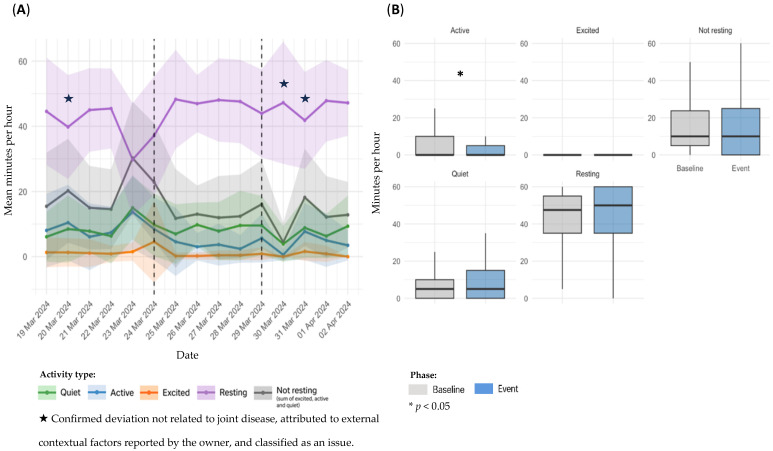
Sensor-derived activity data for D3, referring to Event 4; (**A**): temporal trends in mean daily activity (minutes per hour) ± SD across pre-event, event, and post-event periods; (**B**): comparison of activity metrics between individual baseline and event period.

**Figure 6:**
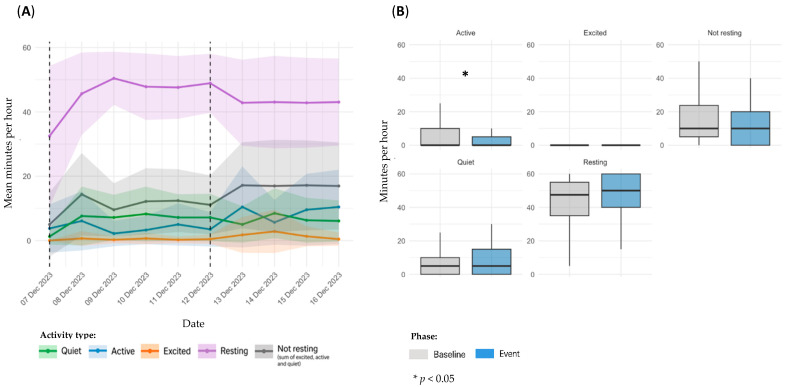
Sensor-derived activity data for D3, referring to Event 5; (**A**): temporal trends in mean daily activity (minutes per hour) ± SD across pre-event, event, and post-event periods; (**B**): comparison of activity metrics between individual baseline and event period.

**Figure 7:**
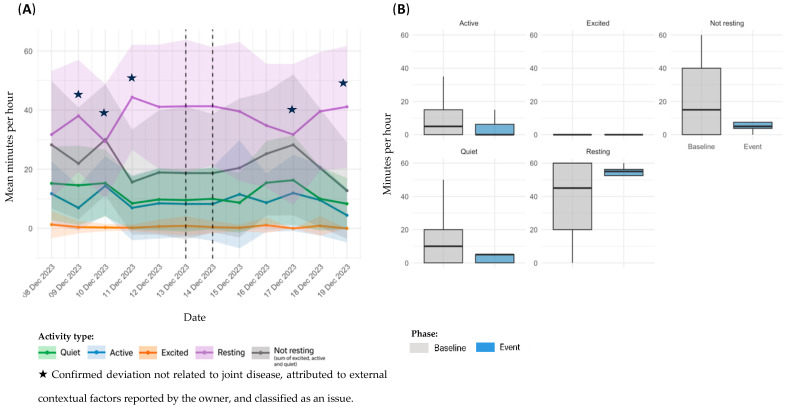
Sensor-derived activity data for D4, referring to Event 6; (**A**): temporal trends in mean daily activity (minutes per hour) ± SD across pre-event, event, and post-event periods; (**B**): comparison of activity metrics between individual baseline and event period.

**Figure 8:**
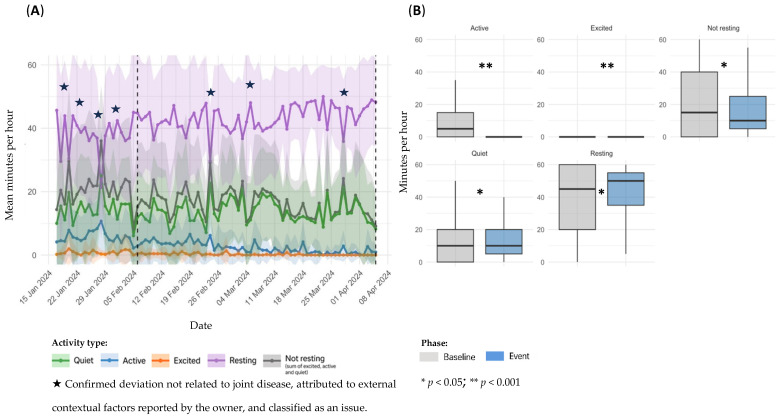
Sensor-derived activity data for D4, referring to Event 7; (**A**): temporal trends in mean daily activity (minutes per hour) ± SD across pre-event, event, and post-event periods; (**B**): comparison of activity metrics between individual baseline and event period.

**Figure 9:**
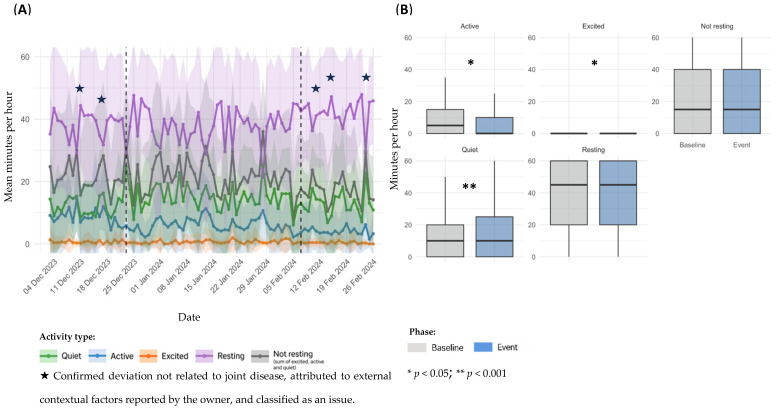
Sensor-derived activity data for D4, referring to Event 8; (**A**): temporal trends in mean daily activity (minutes per hour) ± SD across pre-event, event, and post-event periods; (**B**): comparison of activity metrics between individual baseline and event period.

For each documented event, sensor data were visualized using two complementary plots: a temporal representation of daily activity patterns (A) and a statistical comparison of activity metrics between baseline and event periods (B). Figure 2, Figure 3, Figure 4, Figure 5, Figure 6, Figure 7, Figure 8 and Figure 9 summarize the activity changes observed during each event.

#### 3.4.1. OA Flare-Ups

**Event 1**

Sensor-derived activity data for dog D1, referring to Event 1, are presented in Figure 2. Panel A illustrates temporal trends in daily activity; panel B shows statistical comparisons between the baseline and event periods.

Although Figure 2 displays a 5-day window before and after the event, a gradual decline in activity levels appears to have started approximately 15 days prior to the reported event (additional data available in the Supplementary Materials). In the temporal analysis, a marked increase in *Resting* time and a decrease in overall activity were observed during the event period (Figure 2A). Notably, a modest rise in activity and reduction in *Resting* were noted immediately following the initiation of NSAIDs and gabapentin on February 14. While causality cannot be inferred, this temporal alignment may suggest a clinical response to treatment. In comparison between baseline and event periods, *Excited* activity decreased significantly (mean: 0.375 ± 1.80 vs. 0.0357 min/h; ± 0.423; *p* = 0.0225), although the absolute values were extremely low, with a sparse distribution. No other significant differences were identified, although *Active* time showed a downward trend (5.00 ± 8.19 vs. 3.82 ± 6.91 min/h; *p* = 0.2518). Remaining categories: *Quiet* (9.94 ± 11.5 vs. 10.8 ± 13.8 min/h; *p* = 0.9170), *Not resting* (15.3 ± 17.0 vs. 14.7 ± 17.9 min/h; *p* = 0.5467), and *Resting* (44.1 ± 17.6 vs. 45.2 ± 18.0 min/h; *p* = 0.4785) (Figure 2B).

**Event 2**

Sensor-derived activity data for dog D2, referring to Event 2, are presented in Figure 3. Panel A illustrates temporal trends in daily activity; panel B shows statistical comparisons between the baseline and event periods.

Temporal analysis revealed relatively stable trends across all categories, with only mild daily fluctuations and no evident disruption in overall patterns (Figure 3A). In the comparison between baseline and event periods, no significant differences were identified across any of the activity metrics analyzed: *Quiet* (11.1 ± 12.7 vs. 11.0 ± 11.2 min/h: *p* = 0.5967), *Active* (5.85 ± 8.67 vs. 6.24 ± 8.43 min/h; *p* = 0.4141), *Excited* (2.49 ± 7.49 vs. 1.90 ± 6.62 min/h; *p* = 0.1694), *Resting* (40.2 ± 20.4 vs. 40.8 ± 18.2 min/h; *p* = 0.8719), and *Not resting* (19.4 ± 20.2 vs. 19.2 ± 18.2 min/h; *p* = 0.7401) (Figure 3B).

**Event 3**

Sensor-derived activity data for dog D2, referring to Event 3, are presented in Figure 4. Panel A illustrates temporal trends in daily activity; panel B shows statistical comparisons between the baseline and event periods.

In the temporal analysis, a transient alteration was observed on the day of the event, with a slight increase in *Resting* time and a reduction in activity levels (Figure 4A). This variation rapidly normalized later in the day, returning to patterns consistent with the individual’s baseline. Detailed hourly information of the event that supports these findings is included in the Supplementary File. Comparison between baseline and event periods revealed no significant changes across any activity category: *Quiet* (11.1 ± 12.7 vs. 11.9 ± 15.2 min/h; *p* = 0.9390), *Excited* (2.49 ± 7.49 vs. 3.54 ± 10.7 min/h; *p* = 0.7452), *Resting* (40.2 ± 20.4 vs. 41.2 ± 21.0 min/h; *p* = 0.7694), and *Not resting* (19.4 ± 20.2 vs. 18.8 ± 21.0 min/h; *p* = 0.8259) (Figure 4B). Nonetheless, *Active* time (5.85 ± 8.67 vs. 3.33 ± 5.04 min/h; *p* = 0.2891) demonstrated a trend toward lower values.

**Event 4**

Sensor-derived activity data for dog D3, referring to Event 4, are presented in Figure 5. Panel A illustrates temporal changes before, during, and after the event; panel B presents baseline versus event statistical comparisons.

In the temporal analysis, a marked peak in all activity categories and a pronounced reduction in *Resting* time were observed on 23 March, the day preceding the reported clinical signs, potentially indicating an episode of overexertion. This was followed by a decline in activity levels, particularly in *Active* and *Not resting* categories, with a gradual improvement by 29 March (Figure 5A). Analysis of baseline versus event periods indicated that *Active* levels were reduced during the event (mean: 6.18 ± 8.60 vs. 4.21 ± 7.50 min/h; *p* = 0.0178). *Quiet* (7.01 ± 7.64 vs. 8.64 ± 8.87 min/h; *p* = 0.1243), *Excited* time (1.05 ± 3.53 vs. 1.12 ± 5.72 min/h; *p* = 0.8177) and *Resting* (45.3 ± 13.0 vs. 46.0 ± 14.1 min/h; *p* = 0.3510) displayed an upward trend, while *Not resting* (14.2 ± 12.6 vs. 14.0 ± 14.1 min/h; *p* = 0.5078) (Figure 5B) did not.

**Event 5**

Sensor-derived activity data for dog D3, referring to Event 5, are presented in Figure 6. Panel A illustrates temporal changes before, during, and after the event; panel B presents baseline versus event statistical comparisons.

In the temporal analysis (Figure 6A), sensor data indicated a decrease in overall activity and a concurrent increase in *Resting* time throughout the event period. As the event coincided with the first day of sensor use, a post-stabilization phase was defined and used as the individual baseline for comparison. Following the end of the event, *Active*, *Excited* and *Not resting* time increased while *Resting* values decreased, suggesting a recovery of the dog’s typical activity pattern. A comparison between baseline and event periods revealed a reduction in *Active* time during the event (6.18 ± 8.60 vs. 4.01 ± 6.36 min/h; *p* = 0.0471). Although no significant differences were observed in the remaining categories, *Quiet* (7.01 ± 7.64 vs. 7.18 ± 7.79 min/h; *p* = 0.9048) and *Resting* time (45.3 ± 13.0 vs. 47.5 ± 11.2 min/h; *p* = 0.2095) showed a slight upward trend. In contrast, *Excited* (1.05 ± 3.53 vs. 0.45 ± 1.60 min/h; *p* = 0.2138) and *Not resting* (14.2 ± 12.6 vs. 11.6 ± 10.3 min/h; *p* = 0.1285) tended to decrease during the event period (Figure 6B).

**Event 6**

Sensor-derived activity data for dog D4, referring to Event 6, are presented in Figure 7. Panel A illustrates temporal changes before, during, and after the event; panel B presents baseline versus event statistical comparisons.

Although daily averages did not reflect marked changes on the event day (Figure 7A), hourly data revealed a decrease in activity between 1:00 PM and 6:00 PM, following a surge in high-paced activity earlier that day. These additional data are detailed in the Supplementary File and align with the clinical interpretation of an overexertion episode with same-day resolution. When comparing baseline and event periods, a tendency for reduced activity was observed across all categories, although none of the differences reached statistical significance: *Quiet* (11.8 ± 12.3 vs. 5.83 ± 8.21 min/h; *p* = 0.1231), *Active* (8.43 ± 11.0 vs. 4.58 ± 6.89 min/h; *p* = 0.3099), *Excited* (0.639 ± 2.74 vs. 1.25 ± 4.33 min/h; *p* = 0.8025), *Resting* (38.8 ± 20.8 vs. 48.3 ± 17.5 min/h; *p* = 0.1914), and *Not resting* (20.8 ± 20.6 vs. 11.7 ± 17.5 min/h; *p* = 0.2086) (Figure 7B).

**Event 7**

Sensor-derived activity data for dog D4, referring to Event 7, are presented in Figure 8. Panel A illustrates temporal changes before, during, and after the event; panel B presents baseline versus event statistical comparisons.

Sensor data revealed a progressive decline in overall activity metrics, with reductions in *Active*, *Excited*, and *Not resting* categories. *Resting* time increased in parallel, potentially suggesting chronic discomfort. Despite day-to-day variability, the downward trend in activity levels remained consistent until the end of the study. No recovery was apparent within the monitored period (Figure 8A).

Relative to baseline, the event period was associated with a reduction in *Active* (8.43 ± 11.0 vs. 2.24 ± 5.26 min/h; *p* < 0.001), *Excited* (0.639 ± 2.74 vs. 0.207 ± 1.42 min/h; *p* < 0.001), *Quiet* (11.8 ± 12.3 vs. 13.6 ± 13.5 min/h; *p* = 0.0199), and *Not resting* categories (20.8 ± 20.6 vs. 16.1 ± 16.0 min/h; *p* = 0.0285). *Resting time* showed a corresponding increase (38.8 ± 20.8 vs. 43.7 ± 16.2 min/h; *p* = 0.0224) (Figure 8B).

#### 3.4.2. Other Non-Orthopedic Health Events

**Event 8**

Sensor-derived activity data for dog D4, referring to Event 8, are presented in Figure 9. Panel A illustrates temporal changes before, during, and after the event; panel B presents baseline versus event statistical comparisons.

In the temporal analysis (Figure 9A), the event onset was marked by a noticeable decrease in *Resting* time, along with an increase in *Quiet* and *Not resting* parameters. No consistent deviation emerged following the clinical signs reported on 23 December. Although intermittent fluctuations in activity and *Resting* levels were observed throughout the event period, a marked shift, characterized by reduced activity and increased *Resting*, was noted after the first corticosteroid administration, aligning with the expected pharmacological effect.

Event-phase data, relative to baseline, revealed an increase in *Quiet* time (8.86 ± 10.2 vs. 15.5 ± 14.2 min/h; *p* < 0.001), along with a reduction in *Active* (12.3 ± 13.8 vs. 7.53 ± 10.4 min/h; *p* = 0.0092) and *Excited* activity (3.12 ± 6.83 vs. 1.03 ± 3.53 min/h; *p* = 0.0070). No significant changes were observed in *Resting* (35.3 ± 21.4 vs. 36.0 ± 20.5 min/h; *p* = 0.7568) or *Not resting* (24.3 ± 21.3 vs. 24.0 ± 20.5 min/h; *p* = 0.8612) categories (Figure 9B).

**Event 9**

**Figure 10:**
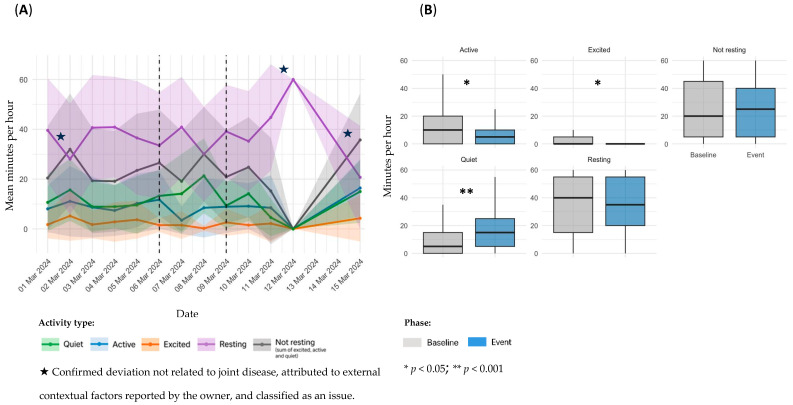
Sensor-derived activity data for D5, referring to Event 9; (**A**): temporal trends in mean daily activity (minutes per hour) ± SD across pre-event, event, and post-event periods; (**B**): comparison of activity metrics between individual baseline and event period.

Sensor-derived activity data for dog D5, referring to Event 9, are presented in Figure 10. Panel A illustrates temporal changes before, during, and after the event; panel B presents baseline versus event statistical comparisons.

Temporal analysis revealed a reduction in *Resting* time on the day of the event, with a concurrent increase in *Quiet* time. A declining trend in *Excited* behavior was also noted (Figure 10A). When compared to the baseline, *Active* time was significantly reduced (12.3 ± 13.8 vs. 5.71 ± 9.30 min/h; *p* = 0.0010), along with *Excited* time (3.12 ± 6.83 vs. 0.816 ± 3.87 min/h; *p* = 0.0022). An increase in *Quiet* activity was also increased (8.86 ± 10.2 vs. 16.8 ± 15.6 min/h; *p* < 0.001). No significant changes were observed in *Not resting* (24.3 ± 21.3 vs. 23.4 ± 20.1 min/h; *p* = 0.7859) or *Resting* (35.3 ± 21.4 vs. 36.0 ± 20.1 min/h; *p* = 0.6993) (Figure 10B).

### 3.5. Survey Results

Assessment survey results from both dog owners and the veterinarian in charge of the cases, focusing on perceived usability, utility, and clinical applicability, are presented in Table 4 and Table 5.

## 4. Discussion

Osteoarthritis is a progressive joint disorder that compromises mobility and quality of life of dogs [1,3]. Clinical signs often fluctuate and may go unnoticed in early or subtle phases, challenging early recognition and consistent monitoring [12,33]. In this exploratory study, a wearable sensor system combining gyroscopic and accelerometric data was used to remotely track daily activity and rest patterns in five dogs with OA providing preliminary insights into its capacity to detect deviations associated with clinical events.

Sensor-derived changes in activity patterns varied across individuals and events, possibly reflecting individual variability and heterogeneity in the clinical expression of OA. Factors such as age, joint pain intensity, muscle atrophy and others have previously been shown to modulate activity profiles in OA dogs [26], which may explain the inter-event variability observed in longer events such as Event 7.

In OA-flare-up events, statistically significant deviations included increased *Resting* time (Event 7) and decreased *Quiet* (Event 7), *Active* (Events 4, 5, 7), and *Excited* activity (Events 1, 7). The absence of statistically significant differences in some events likely reflects their short duration, occasionally limited to a single day (Event 3 and 6). Beyond inferential statistics, visual analysis of temporal trends added interpretative value, suggesting clinically relevant fluctuations not captured by statistical tests alone. In Event 1, a pre-event decline in activity and subsequent improvement after treatment started suggested a pattern consistent with pain expression and therapeutic response. Although this study did not aim to assess treatment efficacy, the observation of deviations temporally aligned with therapeutic intervention in Events 1 and 2 may point to the value of using wearable sensors as continuous and objective outcome measures in clinical trials, complementing standard evaluations with real-time activity data. This perspective has been supported by previous studies, which proposed that automatically generated activity metrics should serve as potential outcome measures in OA dog clinical trials [34], demonstrated that accelerometer-based data can capture increases in physical activity and reductions in analgesic use following low-level laser therapy in dogs with OA [28], and showed that higher initial impairment levels are associated with greater positive changes in activity following NSAID treatment [13].

In short-duration episodes, intra-day fluctuations, such as the transient variations detected in Events 3 and 6, were not clearly reflected in daily trend plots, as they occurred within specific hours. Relying solely on average daily metrics may mask subtle but clinically relevant temporal patterns. Complementing the main analysis, observation of hourly-level data enabled the detection of brief changes that would likely be overlooked by owner reports or conventional clinical assessments. Functional linear modeling (FLM) detected improvements in sleep quality in OA dogs treated with meloxicam, differences that conventional summary statistics fail to reveal [27]. Although we did not use FLM, our findings align with the principle that longitudinal detailed analysis enhances the ability to identify clinically meaningful fluctuations. Furthermore, Lee et al. identified consistent intra-day and intra-week activity patterns in OA dogs, including higher activity levels on weekends, when owners are presumably more available, and two daily morning and afternoon peaks, likely corresponding to feeding, walking, and elimination routines [14]. Although individual routines (e.g., feeding, elimination) were not systematically recorded, and natural activity variability cannot be entirely excluded, the temporal alignment between activity deviations and the onset of clinical signs in single-day events, such as Events 3 and 6, suggests that these fluctuations were more likely associated with the clinical event.

Previous studies have shown that dogs with OA-related pain exhibited measurable changes in overall activity, in particular of high intensity [15]; however, to our knowledge, the hypothesis of wearable sensors to anticipate clinical signs prior to owner recognition remains unexplored in the current literature. In Event 1, a gradual decline in activity and increased *Resting* time were observed prior to symptom recognition, triggering alerts on the monitoring platform. This potential for early detection may be particularly valuable in chronic conditions such as OA, where early manifestations are often subtle, progressive, and easily missed [12]. By identifying early deviations, the system could prompt owner vigilance and facilitate timely therapeutic adjustments. The potential anticipatory capacity observed here may represent a valuable focus for future studies.

The sensor data also contributed to contextual interpretation of some observed fluctuations. In Event 4, an abrupt activity spike followed by a marked decline supported the hypothesis of physical overexertion, in alignment with the owner’s report. Similarly, in Event 6, transient variations identified through hourly analysis were also consistent with an overexertion episode, allowing for an improved understanding of the clinical scenario. Given the weak correlation previously described between clinical and owner-reported pain scores and sensor metrics [14], these insights underscore the value of multimodal monitoring.

Despite these observations of potential clinical utility, certain deviations were not detected by the sensor. In line with Lee et al.’s findings, in Event 2, although clinical signs were reported and a favorable response to anti-inflammatory treatment was observed, activity metrics remained largely stable. This suggests that not all pain-related manifestations could be necessarily associated with detectable deviations in activity patterns. In addition, not all detected deviations corresponded to clinical events, and contextual interpretation was required to distinguish them from non-pathological causes. In all events except Event 5, some deviations identified in the plots were instead associated with external contextual factors reported by the owners, such as fireworks, travel, or social gatherings. These deviations, however, were annotated in the daily trend plots for clarity in data interpretation. One study found a significant but not clinically relevant correlation between daily activity and environmental factors, such as average temperature and daylight hours [35]. Although we did not formally quantify environmental variables, our observations suggest that the contextual influences, including fireworks, travel, and social gatherings, may similarly affect sensor data. While previous studies have rarely addressed the influence of such non-clinical variables on sensor-derived activity data, our observations underscore the value of owner-reported context in interpreting sensor data, particularly in studies conducted in non-controlled, home-based settings.

In longer events (Events 7 and 8), although significant changes were detected between baseline and event periods, intra-event variability in activity patterns was evident, possibly reflecting episodic fluctuations in clinical severity [26], environmental triggers [35], or behavioral changes secondary to chronic pain [3], underscoring the need for contextual interpretation in prolonged episodes characteristic of chronic conditions.

While this study focused primarily on OA, deviations were also detected during non-orthopedic events, which were analyzed in accordance with the study’s exploratory and observational scope. In event 8, a dermatological flare-up, an increase in *Quiet* time and a concurrent reduction in *Active* time were noted, possibly reflecting pruritus. Deviations occurred both at the onset of the event and following therapeutic intervention. While these observations were based solely on general activity metrics, they raise the possibility that pruritus-specific indicators could provide more accurate insights in dermatological contexts. In Event 9, an episode of acute gastroenteritis, reduced *Excited* and *Active* and increased *Quiet* time likely reflected the systemic malaise. Although general activity metrics captured these changes, they did not directly assess important clinical indicators such as food or water intake. Future work could integrate specific metrics such as the quantification of drinking and eating times to enhance clinical interpretation. These observations may support broader applications of wearable sensors beyond OA.

Owner feedback collected during the study provided preliminary insights into the perceived usability of the system. Survey results revealed a generally positive perception, with all participants agreeing that the recorded patterns matched real-life observations. Additionally, 80% reported added value in monitoring long-term trends, and 60% in identifying discomfort. Furthermore, 80% reported that the monitoring process helped them better understand their dog’s physical limits, reflecting an increased awareness of the typical range of activity their dogs were comfortable with or capable of sustaining in daily routines. Moreover, 40% perceived improved follow-up, which they likely associated with the availability of real-time data accessible to the attending veterinarian for clinical monitoring. Although no definitive conclusions can be drawn from such a limited dataset, these exploratory findings offer a complementary perspective on the system’s potential. From the clinical perspective, the veterinarian in charge of case management reported high usability and ease of integration into the clinical workflow. In some cases, monitoring was perceived as more efficient, particularly in maintaining contact with owners. Alerts were considered clinically relevant; however, they did not prompt additional in-person consultations. This outcome likely reflects the study context, where dogs were already under supervision, attending regular rehabilitation sessions and receiving continuous remote follow-up. Still, some alerts prompted occasional follow-up calls or messages to owners. The system influenced diagnosis or treatment in a few cases, and a generally positive impact was perceived on relationship and communication with owners. No change was reported in the veterinarian’s compliance with clinical recommendations.

Overall, these exploratory findings may suggest potential clinical utility, warranting broader investigation.

This exploratory observational study generated preliminary insights into the use of continuous activity tracking for clinical monitoring of dogs diagnosed with OA. However, some limitations must be acknowledged when interpreting these early findings. The final sample size comprised only five dogs, following the exclusion of ten participants based on predefined criteria. This small cohort limits the generalizability of observations and the strength of the inferences drawn from statistical comparisons. Although contextual information from veterinarians and owners was integrated during analysis, residual confounding cannot be fully excluded. Further larger studies will be key to building these preliminary findings and validating the system’s clinical relevance across diverse use cases to account for demographic and breed variability. Moreover, extending the monitoring period could reveal seasonal variations and long-term progression patterns, providing a clearer picture of chronic disease evolution in dogs with OA.

## 5. Conclusions

By continuously tracking activity and rest patterns, the system detected deviations associated with a range of clinical events, including OA flare-ups, as well as other non-orthopedic conditions, that emerged beyond the initial scope of the study. In one event, deviations preceded owner recognition, possibly suggesting the system’s potential for early detection of health changes. However, not all fluctuations corresponded to clinical episodes, and interpretation required contextual input from owners. Sensor-detected changes varied across events, affecting high-paced activity, overall activity, or *Resting* time, underscoring the heterogeneity of physiological responses. Taken together, these preliminary insights may suggest the potential of continuous activity monitoring as a complementary tool in OA management. Further research is warranted to corroborate and expand upon these observations.

## Figures and Tables

**Table 1:** Demographic and clinical characteristics of the five dogs analyzed, including breed, sex and neuter status, age, body weight, total monitoring duration (days), and cumulative hours of valid sensor data.

Dog	Breed	Sex/Neuter Status	Age (Years)	Weight (kg)	Monitoring Duration (Days)	Valid Data (Hours)
D1	Labrador Retriever	Female/Neutered	10	26.9	84	1905
D2	Large Mixed Breed	Male/Neutered	6	30	56	1248
D3	German Shepherd	Male/Intact	7	43	119	2338
D4	Large Mixed Breed	Male/Neutered	12	27.1	126	2873
D5	Jack Russell Terrier	Female/Neutered	7	7	112	2357

**Table 2:** Clinical characterization of study dogs with osteoarthritis.

Dog	Diagnosis	Joints Affected	Severity at Baseline (by COAST/COASTer Stage Methods)	Lameness Grade * (Baseline)	Lameness Grade (Events/End)
D1	OA secondary to hip dysplasia	Bilateral coxofemoral joints	Stage 3 COAST	2	3 (event 1); 2 (end)
D2	OA secondary to hip dysplasia	Bilateral coxofemoral joints	Stage 4 COAST	3	5 (event 2 and 3); 3 (end)
D3	OA secondary to hip dysplasia	Bilateral coxofemoral joints	Stage 3 COAST	3	5 (event 4); 4 (event 5); 3 (end)
D4	OA secondary to immune-mediated polyarthritis caused by ehrlichiosis	Bilateral tarsal joints	Stage 3 COASTer	4	4 (events 6, 7 and 8); 4 (end)
D5	OA	Unilateral right coxofemoral joint	Stage 3 COAST	4	4 (event 9); 4 (end)

Abbreviations: OA = Osteoarthritis; COAST = Canine OsteoArthritis Staging Tool [30]; COASTer = Canine OsteoArthritis Staging Tool excluding radiography [31]. * Lameness grades were assigned using a 0-5 scale, where 0 = no lameness and 5 = predominantly non-weight-bearing [32].

**Table 3:** Overview of documented health events, detailing event classification, dates, duration, and case summaries integrating owner reports and veterinary assessment.

Event Classification	Dog	Event	Event Dates	Duration (Days)	Case Summary
OA flare-ups	D1	1	12–18 February 2024	7	On 12 February, the owner reported abnormal gait, lameness, and neck rigidity via the Maven app. The dog was evaluated by a veterinarian on 14 February, and non-steroidal anti-inflammatory drugs (NSAIDs) and gabapentin were administered for five days.
	D2	2	6–12 March 2024	7	On 6 March, the owner reported lameness. Full recovery was observed by the owner after five days of robenacoxib treatment.
		3	13 February 2024	1	On 13 February, the owner reported limping and abnormal gait. An oral anti-inflammatory was administered around lunchtime, with resolution of clinical signs by the end of the day.
	D3	4	24–29 March 2024	6	On 24 March, the owner reported limping and difficulty rising, following a suspected episode of overexertion. Clinical signs improved after five days of rest and controlled activity.
		5	7–12 December 2023	6	On 7 December, the owner reported intermittent right pelvic limb lameness and mood changes. Same-day veterinary evaluation included laser therapy, acupuncture, and a rest recommendation.
	D4	6	13 December 2023	1	On 13 December, the owner reported limping in the morning and altered gait in the afternoon. Clinical signs resolved on the same day with rest
		7	6 February–4 April 2024	58	From 6 February onwards, the owner reported signs of lethargy. Subsequent veterinary assessment revealed findings consistent with OA of immune-mediated origin (ehrlichiosis), and a 28-day treatment with doxycycline was initiated on 17 March. Clinical signs persisted until the end of the study.
Other non-orthopedic health events		8	23 December–7 February 2024	16	On the morning of 23 December, the owner observed skin lesions on the nose and paws, and a corticosteroid injection was administered later that day. Oclacitinib was initiated on 7 February. The clinical signs were later confirmed as a dermatological flare-up.
	D5	9	6–9 March 2024	4	On 7 March, the owner reported symptoms including elevated body temperature, soft stools, and dull coat. Veterinary evaluation the next day confirmed acute gastroenteritis, and treatment included probiotics and a gastrointestinal diet.

**Table 4:** Survey results on system usage and perceived usability from dog owners.

Owner Responses		
1. Frequency of system use		
Parameter	Round 1 (%)	Round 2 (%)
Daily	80	60
Weekly	20	40
2. Degree of agreement with statements about the system		
Parameter	Round 1 (% Yes)	Round 2 (% Yes)
The system effectively records movement periods	100	100
Events on the dashboard match what I observe in reality.	100	100
The data correspond to my perception of sleep/activity.	100	80
The system is useful to understand my dog’s overall health.	80	60
The system helps identify moments of pain or discomfort.	60	60
Monitoring helps me understand my dog’s activity limits.	80	80
The system improved clinical follow-up by the veterinary team.	40	40
I see added value in long-term monitoring.	80	80
3. System recommendation		
Recommendation likelihood—mean score (0–10 scale) *	7	8

* (0 = Not likely at all, 10 = Extremely likely).

**Table 5:** Feedback from the veterinarian in charge of the cases on system integration and clinical relevance.

Veterinarian Responses	
Question	Response
Familiarity with the Maven application and its capabilities (0–10) *	9
Learned to use and explain the system to clients quickly and intuitively? (Yes/No)	Yes
Familiarity with how all data are collected and presented in the clinic portal (0–10) *	8
Overall ease of use of the portal (0–10) **	10
Specific feature difficult to navigate or understand? (Yes/No)	No
Ease of integrating daily report reading into clinical workflow (Yes/No)	Yes
Who was responsible for reading and making decisions based on the system reports?	Myself
Time spent reading reports/emails and exploring patient data on portal	Some time weekly
Did monitoring become more efficient? If so, to what extent	In some cases, it helped maintain owner contact
Alerts received were clinically relevant? (Yes/No)	Yes
Practicality of information for diagnosis, treatment, and monitoring	Quite practical
Did report changes prompt calls or messages with owners? (Yes/No)	Yes, but only occasionally
Did alerts lead to scheduling new follow-up appointments? If yes, how many on average	No
Owners’ involvement affected relationship with clinical team (Positive/Negative/Neither)	Positively
Cases where system positively influenced diagnosis or treatment plan	In a few cases
Did use of Maven app increase veterinarians’ compliance with clinical recommendations? (Yes/No)	I did not notice
If previous answer positive, which feature is most relevant?	Not applicable
Did data provided help improve communication with owners? (Yes/No)	Yes
Suggestions to improve system’s usability and practical relevance	No

* (0 = not familiar at all; 10 = extremely familiar). ** (0 = extremely difficult to use; 10 = extremely easy to use).

## Data Availability

The original contributions presented in this study are included in the article/Supplementary Materials. Further inquiries can be directed to the corresponding author(s).

## Supplementary Materials

The following supporting information can be downloaded at https://www.mdpi.com/article/10.3390/ani15182639/s1: Table S1: Hourly activity metrics for Dog D1 during the monitoring period; Table S2: Hourly activity metrics for Dog D2 during the monitoring period; Table S3: Hourly activity metrics for Dog D3 during the monitoring period; Table S4: Hourly activity metrics for Dog D4 during the monitoring period; Table S5: Hourly activity metrics for Dog D5 during the monitoring period.

## Abbreviations

The following abbreviations are used in this manuscript:

OA	Osteoarthritis
NSAID(s)	Non-Steroidal Anti-Inflammatory Drug(s)
FLM	Functional Linear Modeling
